# Simultaneous Quantification of Flavonol Glycosides, Terpene Lactones, Biflavones, Proanthocyanidins, and Ginkgolic Acids in *Ginkgo biloba* Leaves from Fruit Cultivars by Ultrahigh-Performance Liquid Chromatography Coupled with Triple Quadrupole Mass Spectrometry

**DOI:** 10.1155/2013/582591

**Published:** 2012-12-27

**Authors:** Xin Yao, Gui-Sheng Zhou, Yu-Ping Tang, Ye-Fei Qian, Han-Liang Guan, Hanqing Pang, Shaoqing Zhu, Xuan Mo, Shu-Lan Su, Chun Jin, Yong Qin, Da-Wei Qian, Jin-Ao Duan

**Affiliations:** ^1^Jiangsu Key Laboratory for High Technology of TCM Formulae Research, Nanjing University of Chinese Medicine, Nanjing 210046, China; ^2^Jiangsu Shenlong Pharmaceutical Co., Ltd., Yancheng, Jiangsu 224200, China

## Abstract

On the basis of liquid chromatography coupled with triple quadrupole mass spectrometry working in multiple reaction monitoring mode, an analytical method has been established to simultaneously determine flavonol glycosides, terpene lactones, biflavones, proanthocyanidins, and ginkgolic acids in *Ginkgo biloba* leaves. Chromatographic separation was carried out on an Acquity BEH C_18_ column (100 mm × 2.1 mm, 1.7 **μ**m) with gradient elution of acetonitrile and 0.10% formic acid (v/v) at a flow rate of 0.4 mL/min, and column temperature 30°C. The developed method was validated in terms of linearity, accuracy, precision, stability, and sensitivity. The optimized method was successfully applied to analyze twenty-two *G. biloba* leaf samples of fruit cultivars collected from different places in China. Furthermore, hierarchical clustering analysis (HCA) was performed to evaluate and classify the samples according to the contents of the twenty-four chemical constituents. All of the results demonstrated that the developed method was useful for the overall evaluation of the quality of *G. biloba* leaves, and this study was also helpful for the comprehensive utilization and development of *G. biloba* resources.

## 1. Introduction

The dried leaves of *Ginkgo biloba *L. have been used as herbal remedies in China, and now their extracts are one of the most widely used herbal products and/or dietary supplements in the world. The products are popular for their alleged tonic effect and possible curative and restorative properties [1–3]. Important constituents in *G. biloba* leaves include flavonol glycosides, terpene lactones, biflavones, proanthocyanidins, and ginkgolic acids, of which the flavonol glycosides, terpene lactones, and proanthocyanidins are considered to be the main components for their beneficial effects. These compound classes have received by far the most attention [1, 4–6]. Flavonol glycosides are widely known for their antioxidant and free radical scavenging activity [7]. Ginkgolides are potent and selective platelet-activating-factor antagonists, and recently there has been considerable interest concerning the antagonistic effect of ginkgolides on the glycine receptor [8–10]. Proanthocyanidins have substantial antioxidant activity and may modulate several reactions involved in cancer processes [11]. In addition, most Gymnospermae plants were characterized mainly by the occurrence of biflavonoids [12], while ginkgolic acids were considered as potentially hazardous constituents in *G. biloba* leaves because they possessed possibly mutagenic [13] and carcinogenic activity [14].

In the past decade, many scientific analytical methods have been studied on the chemical constituents of *G. biloba *leaves, mainly the flavonoids and terpene lactones. HPLC-ELSD has remained the most widely used method for the analysis of terpene lactones [15–17]. To date, various analytical methods have been used to analyze flavonol glycosides that can be classified as two types: the direct assays and the assays after hydrolysis. The general procedure for the quantitative analysis of flavonol glycosides with an acidic hydrolysis is followed by HPLC determination of the resulting aglycones, based on calibration curves of aglycone standards [18–20]. However, the measurement solely of the flavonol aglycones does not preclude the possibility of adulteration of *G. biloba *leaves with pure flavonol aglycones, pure flavonol glycosides, or other plant material containing flavonols or flavonol glycosides. So, it is necessary to analyze intact glycosides for an accurate evaluation of the quality of *G. biloba *leaves. Of course, flavonol glycosides can be analyzed by HPLC with DAD or MS [21, 22]. For saving the time and cost by reducing multiple sample preparations and mobile phases, HPLC-DAD-ELSD was used for simultaneous determination of intact flavonol glycosides and terpene lactones [23]. However, its low sensitivity and uncertainty in peak identification limited its use in analysis. Recent success in the use of liquid chromatography coupled with triple quadrupole mass (UPLC/MS/MS) for characterizing and quantifying a wide variety of compounds in complex samples [24–26] suggested that UPLC/MS/MS might be a good technique for the simultaneous determination of intact glycosides and terpene lactones. In addition, it is possible that other important constituents of *G. biloba *leaves, such as biflavones, proanthocyanidins, and ginkgolic acids could also analyzed simultaneously.

The *Ginkgo* resources are quite rich in China. Most researches on *G. biloba* leaves focus on the leaves collected during July and August from trees 4–7 years old [27–31]; a large number of leaves from fruit cultivars (the tree of above 10 years) are ignored and become obsolete after fruit harvest season (November). Herein, we developed methods for a systematic identification of the main active components in different cultivation sources of *G. biloba* leaves collected in November, which will provide a scientific basis for the comprehensive utilization and development of *G. biloba *resources.

In this paper, by using UPLC/MS/MS, an efficient and sensitive method was developed and validated for simultaneous identification and quantification of twenty-four compounds including flavonol glycosides, terpene lactones, biflavones, proanthocyanidins, and ginkgolic acids in *G. biloba* leaves from fruit cultivars. The proposed method was successfully applied to analyze twenty-two samples collected from different places in China. Furthermore, hierarchical clustering analysis (HCA) was performed to evaluate and classify the samples according to the contents of the twenty-four chemical constituents.

## 2. Experimental

### 2.1. Reagents and Materials

**Figure 1:**
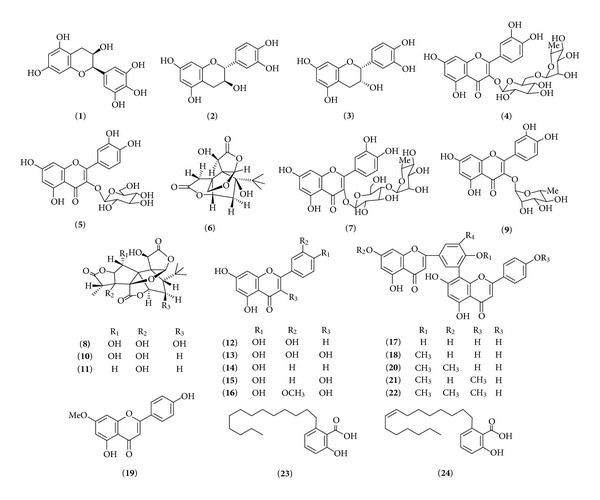
Chemical structures of the investigated target compounds.

Acetonitrile was of HPLC grade from Merck (Darmstadt, Germany), and deionized water (H_2_O) was purified by a superpurification system (Eped Technology Development Co., Ltd., Nanjing, China). Other reagent solutions were of analytical grade (Sinopharm Chemical Reagent Co., Ltd., Shanghai, China). The chemical structures of the reference standards including (−)-epigallocatechin (**1**), (+)-catechin hydrate (**2**), (−)-epicatechin (**3**), quercetin 3-*O*-[6-*O*-(*α*-L-rhamnosyl)-*β*-D-glucoside] (**4**), quercetin 3-*O*-*β*-D-glucoside (**5**), bilobalide (**6**), quercetin 3-*O*-[4-*O*-(*α*-L-rhamnosyl)-*β*-D-glucoside] (**7**), ginkgolide C (**8**), quercetin 3-*O*-*α*-L-rhamnoside (**9**), ginkgolide B (**10**), ginkgolide A (**11**), luteolin (**12**), quercetin (**13**), apigenin (**14**), kaempferol (**15**), isorhamnetin (**16**), amentoflavone (**17**), bilobetin (**18**), genkwanin (**19**), ginkgetin (**20**), isoginkgetin (**21**), sciadopitysin (**22**), ginkgoneolic acid (**23**), and ginkgolic acid (**24**) are shown in Figure 1. Chemical standards of **1**, **2**, and** 3** were purchased from Sigma-Aldrich (St. Louis, MO, USA). Others were all previously isolated and identified from *G. biloba* leaves in our laboratory, and identified by ^1^H-NMR, ^13^C-NMR, MS, and UV spectra. The purity of each compound was >98%, determined by HPLC analysis.

Twenty-two batches of samples from different habitats in China were collected from the trees of 20 years in early November 2011. The detailed information of all samples is listed in Table 1. The botanical origin of the sample was identified by Dr. Hui Yan (Department of Pharmacognosy, Nanjing University of Chinese Medicine, China), and the voucher specimens were deposited at the Herbarium in the Jiangsu Key Laboratory for TCM Formulae Research, Nanjing University of Chinese Medicine, China. After collection, the leaves were dried at 55°C for seven days.

### 2.2. Preparation of Standard Solutions

A mixed standard stock solution containing the twenty-four analytes was prepared in 70% aqueous methanol and then diluted with 70% aqueous methanol to appropriate concentrations for establishing calibration curves. Solutions containing different concentrations of the twenty-four analytes were injected in triplicate, and the calibration curves were plotted by the peak area ratio versus the concentrations of each analyte. The standard solutions were filtered through a 0.22 *μ*m membrane prior to injection.

### 2.3. Sample Preparation

After being dried at 55°C for seven days, the leaves were pulverized to homogenous powders (40 mesh). The dried powder (0.5 g) was weighed accurately into a 100 mL conical flash with a stopper, and 40 mL of 70% aqueous methanol was added. After accurate weighting, ultrasonication (40 kHz) was performed at room temperature for 60 min, and then the same solvent was added to compensate for the weight lost during the extraction. The solution was adequately mixed and followed by centrifugation at 13000 rpm for 10 min. For the quantification of compounds **20**–**24**, the supernatants were diluted 10 or 100 times with 70% aqueous methanol, while for the others, the supernatants did not require dilution. All the solutions were stored at 4°C and filtered through a 0.22 *μ*m membrane filter before injection into the UPLC system for analysis.

### 2.4. Apparatus and Chromatographic Conditions

Determinations were performed on a Waters Acquity UPLC system (Waters, Milford, MA, USA), consisting of a binary solvent delivery system and an autosampler. An ACQUITY UPLC BEH C_18_ (2.1 × 100 mm I.D., 1.7 *μ*m, Waters, Milford, USA) column was used for all the analyses. The mobile phase consisted of A (0.1% formic acid, v/v) and B (acetonitrile) and was used in gradient elution. UPLC linear gradient conditions were: 0–7 min, 90–65% A; 7–11 min, 65–40% A; 11–14 min, 40–0% A; 14–16 min, 0-0% A. The flow rate of the mobile phase was 0.4 mL/min. The column temperature and injection volume were set at 30°C and 1 *μ*L, respectively. All the analyses were operated using MassLynx XS Software.

Mass spectrometry detection was performed using a Xevo Triple Quadrupole MS (Waters Corp., Milford, MA, USA) equipped with an electrospray ionization (ESI) source. The parameters in the source were set as follows: the desolvation gas flow rate set to 1,000 L/h at a temperature of 550°C, the cone gas flow rate set at 50 L/h, and the source temperature at 150°C. The capillary voltage was set to 3,000 V. The analyte detection was performed by using multiple reaction monitoring (MRM). The cone voltage was set depending upon each specific MRM for each compound. Data was collected in MRM mode by screening parent and daughter ions simultaneously. Dwell time was automatically set by the software. All ESI and MS parameters were optimized individually for each target compound and are listed in Table 2.

### 2.5. Hierarchical Cluster Analysis (HCA) of Samples

In order to evaluate the variation of *G. biloba* leaves, HCA was carried out using SPSS 16.0 software. Ward's method was applied, and square Euclidean distance was selected as a measurement. The dendrogram resulted from the 24 investigated compounds' contents derived from UPLC-MS/MS profiles of the tested samples.

### 2.6. Method Validation

A series of analyses such as the linearity, stability, precision, and limits of detection (LOD) and quantification (LOQ) were conducted to validate the performance of the method. The standard solution containing 24 markers was prepared and diluted with 70% aqueous methanol to appropriate concentrations for the construction of calibration curves. Calibration curves were developed by plotting the peak areas versus the corresponding concentrations of each analyte. The precision of the method was evaluated by analyzing the 24 standard compounds. The relative standard deviation (RSD) of peak area was used to evaluate the precision of the developed method. LOD and LOQ for each analyte were determined at the signal-to-noise ratio (*S*/*N*) of about 3 and 10, respectively. The recovery test was used to evaluate the accuracy of this method. The test was performed by adding the corresponding marker compounds at low (80% of the known amounts), medium (same as the known amounts), and high (120% of the known amounts) to the* G. biloba* leaves sample, which had previously been analyzed. The mixture was extracted and analyzed using the aforementioned method in triplicate.

## 3. Result and Discussion

### 3.1. Optimization of Extraction Procedure

To obtain the best extraction conditions, three important factors, namely, extraction methods, extraction solvent, and extraction time, were optimized. The results suggested that ultrasonic extraction was better than reflux extraction, and it had the advantage of greater convenience. Using the sonication method, various solvents including water and 30, 50, 70, and 100% methanol were screened. The best solvent was 70% aqueous methanol, which resulted in fewer interfering peaks and provided the highest values for the contents of the 24 markers. In addition, the extraction time was optimized to 60 min. Each sample extracted with 40 mL was adequate. Finally, suitable extraction conditions were optimized as follows. Each sample was extracted by sonication with 40 mL of 70% aqueous methanol for 60 min. All of the samples were extracted at room temperature.

### 3.2. Optimization of the Chromatographic Conditions

The chromatographic conditions such as column, mobile phase, and gradient elution were optimized in the preliminary test to achieve chromatograms with short analysis time and peak shape without excessive peak tailing. Two analytical columns, Acquity HSS T3 column (100 mm × 2.1 mm, 1.8 *μ*m) and Acquity BEH C_18_ column (100 mm × 2.1 mm, 1.7 *μ*m), were compared, showing that with the latter one obtains chromatograms with better resolution than the former with the same mobile phase. Although all compounds could be separated satisfactorily with the Acquity HSS T3, the chromatographic peak is asymmetric. Therefore, the Acquity BEH C_18_ column was selected as the analytical column. Furthermore, various mixtures of water and methanol were used as the mobile phase but no satisfactory separation was achieved. However, when the mobile phase composed of methanol-water was replaced by acetonitrile-water, the separation resolution was greatly improved. Addition of formic acid to the mobile phase was found to enhance the resolution of target compounds at a flow rate of 0.4 mL/min. The separation was better when column temperature was kept at 30°C rather than 25 and 35°C.

**Figure 2:**
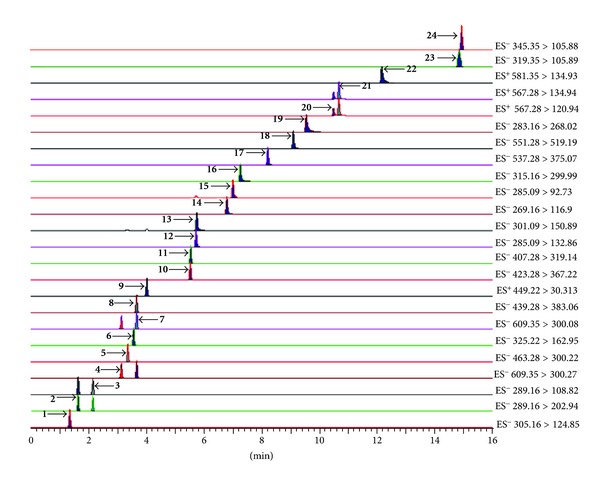
UPLC-MS/MS multiple-reaction monitoring (MRM) chromatograms of 24 analytes.

To select a proper transition for the MS/MS detection of the analyte, all the compounds were examined separately in direct infusion mode and at least two precursor/product ion pairs for each analyte were presented in this study. And then, according to the quantitative results, the highest sensitive and specific ion pairs were selected for the MRM determination. Once the most appropriate precursor/product ion pairs had been determined, the values of cone voltage and collision energy were optimized using the IntelliStart software. All the MRM transitions and parameters applied in the study are listed in Table 2. Representative chromatograms for 24 analytes are shown in Figure 2. The results indicated that the MRM mode has more advantages in the quantification of low content analysis and separation of overlapped constituents in complicated mixtures.

### 3.3. Method Validation

#### 3.3.1. Calibration Curves, LOD, and LOQ

The linearity of the method was obtained by plotting the peak areas versus the corresponding concentrations of each analyte. Each calibration curve was constructed with at least six appropriate concentrations in duplicate. All calibration curves showed good linearity (*r* ^2^ > 0.9903) within the test ranges. LOD and LOQ for each analyte were determined at the signal-to-noise ratio (*S*/*N*) of about 3 and 10, respectively. The overall LODs and LOQs were in the range of 0.04–3.50 ng/mL and 0.10–11.20 ng/mL, respectively. The results are reported in Table 3.

#### 3.3.2. Precision, Repeatability, and Stability

The precision of the method was evaluated by analyzing the 24 standard compounds. The intra- and interday precisions were examined for the mixed standards six times on the same day and the experiments were repeated on three consecutive days, respectively. The relative standard deviation (RSD) was taken as a measure of precision. The RSD values of intra- and interday variations of the 24 analytes were in the range of 1.91%–6.28% and 2.30%–6.30%, respectively. The results are shown in Table 4.

The reproducibility was assessed by analyzing six independently prepared samples using the same method and one of the sample solutions was stored at 20°C and analyzed at 0, 2, 4, 8, 12, and 24 h, respectively, to evaluate the solution's stability. The repeatability and stability present as RSD [32, 33] were in the range from 3.12% to 6.19% and from 0.76% to 5.68%. The results are shown in Table 4. 

A recovery test was used to evaluate the accuracy of this method. The test was performed by adding the corresponding marker compounds at low (80% of the known amounts), medium (same as the known amounts), and high (120% of the known amounts) to a* G. biloba* leaf sample which had previously been analyzed. The mixture was extracted and analyzed using the aforementioned method in triplicate. The average recoveries were estimated by the following formula: recovery  (%) = [(amount found − original amount)/amount added] × 100%. The overall recoveries laid between 94.1% and 104.9% with RSD between 2.66% and 5.81%. The results are shown in Table 4.

### 3.4. Identification and Quantification

Identification of the target peaks was performed by comparing their UPLC retention times and mass/charge ratios (*m*/*z*) with those of the standards. In order to further confirm the structures of the constituents, standards and samples were analyzed by UPLC-MS/MS. Quantification was performed using linear calibration plots of peak areas and concentration.

### 3.5. Sample Analysis

To evaluate the effectiveness of the method, it was applied to the analysis of* G. biloba* leaves collected from the major production areas of *G. biloba* in China (Jiangsu and Guangxi provinces). Due to the fact that the contents of compounds** 20–24** are equal to or greater than two orders of magnitude of the other target compounds in most samples, their quantification was performed by diluting the sample solutions as described in the experimental section before analysis. All the contents are summarized in Table 5. The results showed that the twenty-four constituents were present in most samples. This outcome proved the necessity of the simultaneous determination of these components in order to improve the quality control of this traditional Chinese medicine.

Among these compounds, biflavones, terpene lactones, and ginkgolic acids were dominant components, which were higher than the detected flavonol glycosides and proanthocyanidins. Remarkable differences were found among the contents of the 24 target compounds in *G. biloba* leaves of different cultivation regions. Components **22** and **24 **were dominant in most samples with contents that ranged 2.28–4.92‰ and 0.87–3.00‰, respectively. The contents of total flavonoids, terpene lactones, biflavones, proanthocyanidins, and ginkgolic acids in the analysed samples varied from 670.0 to 3524.7 *μ*g/g, 362.8 to 3668.3 *μ*g/g, 4072.1 to 8641.3 *μ*g/g, 16.9 to 525.1 *μ*g/g, and 1441.5 to 3889.7 *μ*g/g, which should result in differences in their therapeutic potential. Therefore, these active components should also be quantified so as to reflect the therapeutic effect.

**Figure 3:**
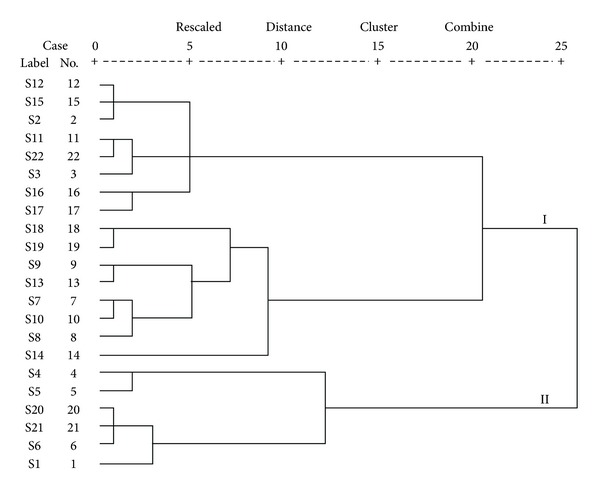
Dendrograms of hierarchical cluster analysis for twenty-two samples of *G. biloba* leaves from different origins.

**Figure 4:**
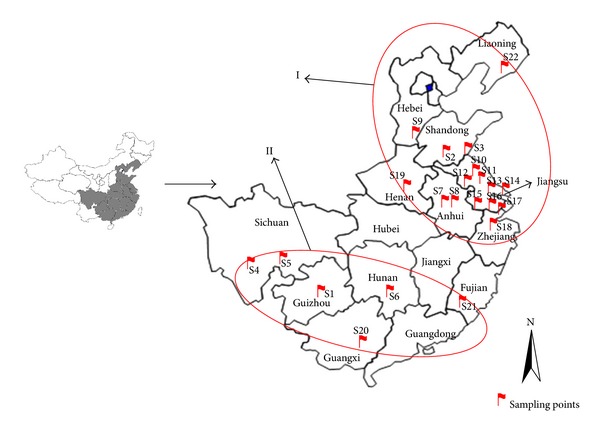
Regional disparity revealed by HCA and location of sampling points on a Chinese map.

Ward's method, which is efficient for the analysis of variance between clusters, was applied, and the square Euclidean distance was selected as a measure of similarity. The dendrogram (Figure 3) shows that the twenty-two tested samples of *G. biloba* leaves were divided into two main clusters (I and II) according to their contents. Samples 1, 4, 5, 6, 20, and 21 were in cluster II and the other samples were in cluster I. The samples with similar chemical profiles were classified into one group. It was noticeable that the distribution of the 24 target compounds in *G. biloba* leaves exhibited regional disparity. In the dendrogram, the northern and southern parts of China were described as cluster I and II, which indicates that the profiles of flavonol glycosides, terpene lactones, biflavones, proanthocyanidins, and ginkgolic acids are similar when they are cultured under the same conditions. The schematic diagram of regional disparity revealed by HCA on the Chinese map is shown in Figure 4.

## 4. Conclusions 

In this study, a reliable, simple, and sensitive method was developed for the identification and quantification of 24 compounds in *G. biloba* leaves by using UPLC/MS/MS. To our knowledge, it is the first time that the UPLC-MS/MS method had been employed to analyze simultaneously twenty-four main bioactive components in *G. biloba* leaves. In this study, the method was applied to investigate *G. biloba* leaves of fruit cultivars of different national regions. The results demonstrate that the developed method is useful for overall evaluation of the quality of *G. biloba* leaves, and this study should be helpful for the comprehensive utilization and development of *G. biloba *resources.

## Figures and Tables

**Table 1:** Cultivation regions of 22 *G. biloba* leaves.

Sample number	Cultivation region
S.1	Guiyang, Guizhou
S.2	Tancheng, Shandong
S.3	Taian, Shandong
S.4	Tainan, Sichuan
S.5	Chengdu, Sichuan
S.6	Yongzhou, Hunan
S.7	Ningguo, Anhui
S.8	Hefei, Anhui
S.9	Shijiazhuang, Hebei
S.10	Lianyungang, Jiangsu
S.11	Taixin, Jiangsu
S.12	Nantong, Jiangsu
S.13	Nanjing, Jiangsu
S.14	Yangzhou, Jiangsu
S.15	Suzhou, Jiangsu
S.16	Pizhou, Jiangsu
S.17	Xuzhou, Jiangsu
S.18	Anji, Zhejiang
S.19	Luoyang, Henan
S.20	Lingchuan, Guangxi
S.21	Changting, Fujian
S.22	Dandong, Liaoning

**Table 2:** The molecular weight (MW), MRM transitions, cone voltage, collision energies, retention times (Rt), and ion mode of 24 target compounds.

Compounds	MW	MRM transitions	Cone voltage (V)	Collision energies (eV)	Rt (min)	Ion mode
**1**	306	305.16 > 124.85	28	20	1.34	ES^−^
**2**	290	289.16 > 202.94	32	20	1.64	ES^−^
**3**	290	289.16 > 108.82	32	26	2.14	ES^−^
**4**	610	609.35 > 300.27	52	34	3.16	ES^−^
**5**	464	463.28 > 300.22	38	28	3.38	ES^−^
**6**	326	325.22 > 162.95	18	20	3.59	ES^−^
**7**	610	609.35 > 300.08	50	34	3.70	ES^−^
**8**	440	439.28 > 383.06	24	14	3.68	ES^−^
**9**	448	449.22 > 30.313	12	12	4.05	ES^+^
**10**	424	423.28 > 367.22	24	14	5.56	ES^−^
**11**	408	407.28 > 319.14	30	14	5.58	ES^−^
**12**	286	285.09 > 132.86	44	32	5.77	ES^−^
**13**	302	301.09 > 150.89	36	22	5.79	ES^−^
**14**	270	269.16 > 116.90	40	34	6.83	ES^−^
**15**	286	285.09 > 92.73	48	34	7.05	ES^−^
**16**	316	315.16 > 299.99	38	22	7.31	ES^−^
**17**	538	537.28 > 375.07	48	40	8.25	ES^−^
**18**	552	551.28 > 519.19	52	30	9.14	ES^−^
**19**	284	283.16 > 268.02	36	26	9.62	ES^−^
**20**	566	567.28 > 120.94	70	44	10.54	ES^+^
**21**	566	567.28 > 134.94	70	44	10.71	ES^+^
**22**	580	581.35 > 134.93	68	44	12.24	ES^+^
**23**	320	319.35 > 105.89	38	36	14.91	ES^−^
**24**	346	345.35 > 105.88	38	44	14.96	ES^−^

**Table 3:** Linear regression data and validation of the developed method for 24 investigated compounds in *G. biloba* leaves.

Analytes	Linear regression data			LOD (ng/mL)	LOQ (ng/mL)
	Regression equation	*R* ^2^	Linear range (*μ*g/mL)		
**1**	*y* = 2741.8*x* + 9.3	0.9999	0.02–16.00	1.36	4.01
**2**	*y* = 2017.6*x* − 59.4	0.9999	0.02–12.40	0.40	1.30
**3**	*y* = 1413.8*x* − 48.2	0.9996	0.02–16.10	0.59	1.98
**4**	*y* = 16288.4*x* − 425.7	0.9999	0.01–24.80	0.11	0.36
**5**	*y* = 13035.1*x* + 502.1	0.9997	0.01–15.30	0.75	2.28
**6**	*y* = 12396.4*x* + 1151.5	0.9995	0.01–39.20	0.20	0.65
**7**	*y* = 19595.7*x* + 2301.8	0.9996	0.01–14.80	0.11	0.35
**8**	*y* = 8072.4*x* + 2614.6	0.9958	0.01–38.40	0.15	0.47
**9**	*y* = 8538.6*x* + 614.3	0.9995	0.01–5.20	0.14	0.47
**10**	*y* = 12519.5*x* + 7421.4	0.9903	0.01–23.60	0.04	0.13
**11**	*y* = 1782.3*x* + 752.6	0.9975	0.02–33.20	0.40	1.35
**12**	*y* = 26238.7*x* + 2228.9	0.9982	0.01–4.20	2.80	8.52
**13**	*y* = 9693.8*x* + 2468.7	0.9952	0.02–4.70	1.26	3.68
**14**	*y* = 14900.4*x* + 773.9	0.9994	0.01–4.12	0.20	0.66
**15**	*y* = 2466.5*x* + 607.2	0.9985	0.02–4.30	2.56	7.60
**16**	*y* = 27430.1*x* − 1049.6	0.9989	0.02–4.35	0.80	2.47
**17**	*y* = 21921.4*x* + 4997.8	0.9951	0.02–13.20	0.65	1.83
**18**	*y* = 11839.7*x* + 10793.6	0.9971	0.05–23.60	0.33	1.02
**19**	*y* = 28959.2*x* + 2819.2	0.9984	0.04–18.90	3.50	10.70
**20**	*y* = 5535.3*x* + 2104.1	0.9935	0.09–18.60	1.20	3.71
**21**	*y* = 10386.1*x* + 6123.9	0.9963	0.10–21.00	0.40	1.33
**22**	*y* = 11892.2*x* + 12144.9	0.9959	0.06–16.00	0.33	1.06
**23**	*y* = 6383.2*x* + 45230.4	0.9985	0.01–20.80	0.04	0.13
**24**	*y* = 1455.5*x* + 4698.7	0.9953	0.02–17.60	0.04	0.13

**Table 4:** Precision, repeatability, stability, and recovery of the 24 target compounds.

Analytes	Precision (RSD, %)				Recovery (%, *n* = 3)	
	Intraday (*n* = 6)	Interday (*n* = 6)	Repeatability (RSD, %, *n* = 6)	Stability (RSD, %, *n* = 6)	Mean	RSD (%)
**1**	3.40	3.66	4.56	3.40	104.3	5.33
**2**	1.91	2.30	6.19	5.68	97.8	5.57
**3**	4.60	4.82	4.12	2.68	95.7	4.71
**4**	2.28	2.56	3.12	2.29	95.6	5.81
**5**	6.28	6.30	4.05	4.29	97.4	4.56
**6**	3.46	3.63	3.76	2.11	104.5	4.50
**7**	3.70	3.86	4.71	1.78	95.6	3.68
**8**	2.58	3.01	5.07	2.76	98.4	2.66
**9**	2.10	2.32	5.12	4.67	98.8	2.98
**10**	2.27	2.50	3.24	2.00	97.9	3.68
**11**	2.36	2.63	5.69	2.99	96.7	3.58
**12**	4.20	4.36	4.17	4.79	98.5	5.04
**13**	2.80	3.01	4.66	3.78	96.6	4.68
**14**	4.09	4.32	3.69	5.46	104.9	4.78
**15**	3.89	4.11	4.12	2.68	103.8	4.21
**16**	3.67	3.80	3.88	2.95	97.6	5.06
**17**	2.78	2.89	3.63	2.74	96.6	3.84
**18**	2.59	2.65	3.87	4.11	103.7	3.22
**19**	3.76	3.88	3.49	4.19	103.3	4.45
**20**	4.44	4.86	5.21	0.76	102.9	4.51
**21**	3.23	3.65	3.69	3.51	98.6	3.08
**22**	3.12	3.71	5.31	3.49	94.1	3.69
**23**	2.53	2.67	5.66	3.16	96.7	4.06
**24**	2.93	3.16	5.47	4.61	102.8	3.01

**Table 5:** The contents of 24 target compounds in *G. biloba* leaves.

S^a^	Contents of analytes (*μ*g/g)																							
	**1**	**2**	**3**	**4**	**5**	**6**	**7**	**8**	**9**	**10**	**11**	**12**	**13**	**14**	**15**	**16**	**17**	**18**	**19**	**20**	**21**	**22**	**23**	**24**
S.1	104.5	46.9	31.7	257.0	42.7	225.1	119.3	381.4	38.6	347.6	143.6	3.1	25.1	10.4	5.3	3.3	58.9	235.6	96.4	853.9	374.4	2549.2	865.9	2842.2
S.2	44.3	145.3	55.7	626.2	99.9	333.6	139.9	394.3	13.7	432.0	160.3	5.1	34.0	45.5	29.2	30.3	121.3	535.0	218.4	1297.1	678.1	3003.9	454.7	1055.9
S.3	106.1	346.8	72.2	866.1	406.6	410.8	84.5	608.0	11.1	479.5	250.4	8.3	27.8	53.7	29.7	34.2	379.8	1002.1	240.8	1280.7	1254.6	3396.5	506.6	1610.5
S.4	133.5	240.9	57.6	618.1	192.3	393.4	247.2	481.1	35.0	390.5	289.8	4.5	2.5	16.1	0.9	4.0	213.0	638.6	63.5	1835.8	821.9	4012.8	710.6	2528.9
S.5	31.8	24.7	14.2	346.2	72.6	95.6	396.3	535.1	44.0	458.8	215.2	16.3	30.2	74.3	30.7	9.5	226.9	671.4	31.2	1917.8	904.9	4920.4	883.7	3005.9
S.6	188.5	33.7	27.7	222.1	36.1	422.0	144.9	501.3	62.7	593.6	135.4	14.7	9.3	29.5	10.9	4.6	184.6	415.7	27.1	1007.5	890.0	3538.8	527.1	2796.9
S.7	124.0	68.7	33.6	439.7	71.5	460.6	49.6	292.8	22.9	276.5	68.3	11.5	5.2	18.9	8.2	3.8	58.6	261.4	62.0	940.7	492.2	3444.4	488.2	1816.0
S.8	+^b^	13.0	3.8	103.8	29.4	37.5	49.7	107.2	4.9	185.0	33.1	2.8	7.5	21.7	8.2	13.2	103.4	428.9	74.3	1285.2	770.3	4333.5	424.0	1199.2
S.9	80.5	91.5	85.5	810.5	174.6	242.4	1.4	404.1	+	372.5	149.9	8.8	9.8	48.0	12.3	9.2	181.0	559.9	58.9	1463.8	730.8	4145.4	485.0	1197.6
S.10	44.2	64.7	28.7	216.5	52.9	202.5	60.8	150.0	30.0	161.9	99.5	+	1.7	4.7	1.4	1.2	130.9	392.6	54.7	1287.2	597.6	3646.3	562.9	1512.7
S.11	162.8	188.3	56.3	527.9	126.9	247.6	66.1	410.5	12.7	382.6	124.7	2.5	3.0	10.5	3.0	4.2	230.7	611.6	50.2	1156.8	826.3	2880.3	640.5	1698.4
S.12	+	26.0	1.0	121.9	47.4	81.5	24.2	309.8	3.4	247.2	62.4	3.7	7.2	24.4	8.7	7.4	205.4	709.9	18.6	1093.4	905.6	2698.9	335.0	1106.5
S.13	37.7	149.0	82.2	868.5	183.6	341.9	249.8	609.7	47.1	455.2	285.3	3.8	2.7	21.8	6.8	4.3	330.1	943.3	44.4	1890.7	896.6	4470.9	379.1	1401.8
S.14	82.7	127.9	62.1	2043.4	641.4	234.0	215.3	228.5	28.3	283.7	90.2	3.9	20.2	16.7	11.2	7.3	148.8	536.9	148.7	1178.1	837.5	3765.6	371.3	874.2
S.15	29.7	46.2	19.3	360.9	82.4	84.8	50.0	226.0	9.9	236.7	42.3	4.6	3.2	26.7	7.4	5.0	118.7	489.6	19.0	953.5	750.8	2660.8	379.7	1386.4
S.16	130.8	139.0	96.9	517.6	113.1	351.3	152.8	848.3	60.3	741.3	364.7	2.8	7.1	8.5	24.1	2.2	74.0	339.8	170.1	556.8	755.9	2445.7	335.9	1173.7
S.17	41.7	35.8	24.3	837.9	131.8	492.9	111.2	596.8	44.9	635.0	261.9	13.6	23.4	53.8	25.6	30.1	165.6	607.8	96.9	464.6	1267.2	2287.7	593.1	1845.5
S.18	115.6	111.4	37.0	419.6	101.0	834.4	156.6	939.9	24.6	774.3	390.1	11.7	15.7	29.9	13.7	10.3	82.3	434.1	69.2	1259.9	654.6	3885.8	340.6	1184.1
S.19	71.7	168.0	54.9	445.6	144.0	1133.2	61.1	1106.6	9.2	802.6	625.9	5.1	14.5	35.9	21.6	21.7	173.6	459.5	117.6	1099.4	761.3	4158.5	403.9	1175.0
S.20	102.8	33.0	17.6	481.2	65.3	553.1	59.9	750.0	16.6	624.2	353.3	1.9	2.8	18.4	2.8	2.7	98.2	318.2	27.7	1016.4	562.5	3185.6	595.1	2475.4
S.21	124.9	43.4	21.7	280.4	83.5	601.2	252.8	534.6	37.7	715.7	395.7	12.3	33.4	29.7	53.7	26.0	141.3	408.0	29.2	942.4	795.0	3300.2	737.1	2287.9
S.22	215.6	170.6	62.3	826.0	463.5	371.8	231.0	531.2	40.4	502.3	262.6	2.2	9.6	11.4	7.1	2.5	181.2	600.1	94.1	1000.7	926.8	3271.3	476.8	1943.6

^a^Sample number. ^b^Below the limit of quantitation.
